# Situation of Diabetes and Related Disease Surveillance in Rural Areas of Jilin Province, Northeast China

**DOI:** 10.3390/ijerph13060538

**Published:** 2016-05-27

**Authors:** Rui Wang, Peng Zhang, Xin Lv, Lingling Jiang, Chunshi Gao, Yuanyuan Song, Yaqin Yu, Bo Li

**Affiliations:** Department of Epidemiology and Biostatistics, School of Public Health, Jilin University, 1163 Xinmin Street, Changchun 130021, Jilin, China; ruiwang14@mails.jlu.edu.cn (R.W.); pengzhang14@mails.jlu.edu.cn (P.Z.); jdhkl_lx@163.com (X.L.); jianglingling.2008@163.com (L.J.); jlugcs@126.com (C.G.); songyuanmei00@163.com (Y.S.); peng14zhang@sina.com (Y.Y.)

**Keywords:** diabetes mellitus, awareness, rural areas, associated factors, disease surveillance

## Abstract

*Background:* Several studies have investigated the prevalence and awareness of diabetes mellitus (DM) in China, but little is known about the situation of DM in the northeastern rural areas. Our present study investigated the prevalence, awareness and associated characteristics of DM in rural areas of Jilin Province, aiming to suggest more efforts for the prevention and control of DM. *Methods:* A multistage stratified random cluster sampling design was used in this cross-sectional study which took place in 2012. Data were collected by face-to-face interviews and physical examinations. Rao-Scott Chi-square test, *t* test and multivariate logistic regression analysis were used. *Results:* The estimated prevalence of DM in rural areas of Jilin province was 7.2%. DM was positively associated with age, Body mass index (BMI), hypotension, dyslipidemia and was high in participants with a family history of diabetes and those who exercise frequently, but low for high education level and married participants. 69.0% participants with DM were aware of their diabetes status, 88.2% of whom received treatment and 34.4% of whom had received treatment controlled their DM status. *Conclusions:* We observed a high prevalence and low awareness status of DM among the rural residents in Jilin Province, but the rate of effective control in those who have received treatment was considerable. The low rate of disease surveillance should draw health authority’s attention.

## 1. Introduction

Diabetes mellitus (DM) is an increasingly common chronic disease that has a great effect not only in regard to clinical effects, but also in regard to economic burden worldwide. According to the International Diabetes Federation (IDF) statistics, there were 382 million people with diabetes in 2013, a number surpassing its earlier predictions. More than 60% of the people with diabetes live in Asia, with almost one-half in China and India combined. The global number of patients with diabetes will increase to 552 million by 2030 [1,2], and the global health expenditure on diabetes is expected to total at least USD 490 billion or ID 561 billion in 2030 [3]. China is a great agricultural country, and more than 900 million people reside in rural areas. Geographical and regional economic limitations seriously affect the health care security in rural areas. As a result, Chinese government has provided a new form of medical system called New Rural Cooperative Medical System (NCMS) to ensure the medical care for residents in rural areas [4,5], but the prevalences of several chronic diseases in rural areas are still higher than that in cities [6]. There is no doubt that the high prevalence and low awareness of DM will aggravate the burden on the Chinese medical system.

In 2010, the overall prevalence of diabetes was estimated to be 11.6% in the Chinese adult population, and 10.3% in rural adult population [7]. Jiangsu Province, which is located in southeast China, reported DM prevalence, awareness, treatment and control rates 7.31%, 58.35%, 51.87% and 14.12%, respectively [8]. However, there are limited data concentrated on the status of DM and related disease surveillance till recently. Our present study was to investigate the prevalence and associated characteristics of DM in rural areas of Jilin Province, northeast China. It is a part of the Project on Present Situation and Change Forecast of Disease Spectrum in Jilin Province of China. We aimed to suggest more efforts for the prevention and control the development of diabetes in rural areas of China. Strengthen grassroots health care services system and conduct regular disease surveillance will achieve a remarkable medical economics in preventing and treating chronic diseases.

## 2. Materials and Methods

### 2.1. Study Design and Population

Data of this study were acquired from the Project on Present Situation and Change Forecast of Disease Spectrum in Jilin Province of China. This face-to-face cross-sectional survey was conducted in 2012 among the general population of Jilin Province aged from 18 to 79 years. A multistage stratified cluster sampling method was used to select all the subjects. The details are as follows: in the first stage, nine administrative regions were selected which covered the whole Jilin Province (Changchun, Jilin City, Siping, Liaoyuan, Tonghua, Baishan, Songyuan, Baicheng, and Yanbian). All these regions are largely responsible for health care delivery. In the second stage, from each of the nine regions, we randomly selected clusters of four districts or countries according to probability proportional to size (PPS) sampling. According to the National Bureau of Statistics of China, we divided each selected districts or counties in this survey into urban and rural areas [9]. Thereafter, we randomly selected four or five communities from the urban and rural strata by PPS. Finally, one adult was randomly selected from each household of the communities mentioned above [6]. In total, people from 32 districts or counties, 95 towns or communities, and 45 units in Jilin Province were selected. We finally recruited 23,050 adult participants, and 21,435 participants completed the survey (response rate: 84.9%). A total of 9600 (44.8%) rural adults were included in the study.

### 2.2. Ethics Approval

This study was approved by the Ethics Committee of Jilin University School of Public Health, and written informed consents were obtained from all the subjects in the survey (Reference Number: 2012-R-011).

### 2.3. Data Collection

Trained investigators (116) conducted the survey using a structured questionnaire after pre-investigation. The questionnaire included the information of participants’ socio-demographic characteristics and other related information on health. In order to ascertain the validity, each questionnaire has been examined by the interviewer after participants completed. Physical examination was conducted by trained investigators for all the participants, anthropometric measurements including height, weight, fasting blood glucose, blood pressure and blood lipid. The Bai Ankang fingertip blood glucose monitor machine (Bayer, Beijing, China) was used to measure fasting blood glucose (FBG) level by taking a small drop of blood from a finger onto a strip of paper. Blood samples were collected from all participants in the morning, after an overnight fast of 10 h or more. After the fieldwork, all data were processed by parallel double entry. Three verifications were carried out to check for incomplete or inconsistent responses, and then deleted the missing data that cannot be repaired.

### 2.4. Definition of Major Variables

The dependent variable in this study was DM, which was defined as a FBG ≥ 7.0 mmol/L or self-reported use of anti-diabetic medications during the 2 weeks prior to the examination. We defined participants who meet the standards for DM or reported themselves had previous diagnosis of diabetes by a medical doctor in a hospital above the country level as diagnosis of diabetes. Participants who reported having a previous diagnosis of diabetes by medical doctors were considered to be aware of their diabetes status (awareness), and those who reported using anti-diabetic medications and other treatments (including lifestyle modification such as physical exercise and low fat diets) during 2 weeks before the examination were defined as under treatment.

According to the grading standards for Chinese adult [10], BMI was calculated as weight (kg)/height (m^2^): 24 ≤ BMI < 28 is overweight; BMI ≥ 28 is obesity and BMI < 18.5 is underweight. According to the criteria of age classification WHO reported in 2012, we divided age range into three groups: young (18–44 years), middle (44–59 years) and old (>60 years) [11]. Smoker was defined as a person who smoked at least one cigarette per day in the past 30 days, and drinker was a person who consumed more than one alcoholic drink per week, included any forms of alcohol. Participants who were classified as “sometimes exercise” were participants who exercised one or two times a week; those who exercised more than three times a week were defined as “exercise frequently”; while those don’t or seldom exercise were defined as “never or rarely exercise”. In addition, the diagnosis of dyslipidemia is based on the presence of one or more of the following criteria: high TG (triglyceride) ≥ 1.70 mmol/L levels, high TC (cholesterin) ≥ 5.18 mmol/L, high LDL-C (low density lipoprotein-cholesterol) ≥ 2.60 mmol/L and low HDL-C (high density lipoprotein cholesterol) ≤1.04 mmol/L for men and ≤1.30 mmol/L for women. Hypertension was defined as systolic and diastolic blood pressure ≥140 mmHg and ≥90 mmHg, respectively, and/or current treatment with antihypertensive medications [12].

### 2.5. Statistical Analyses

Data were analyzed using the SPSS (ver. 22.0; IBM Corp.: Armonk, NY, USA) based on the complex sampling design. Post-stratification adjustment was also carried out to make the sample representative of the population in Jilin Province. The adjustment was made based on the distribution of gender and age groups (18–24 years, 25–34 years, 35–44 years, 45–54 years, 55–64 years, and 65–79 years) in the census of the adult population of Jilin Province in 2010 [6]. Continuous variables were expressed by mean and standard deviation and categorical variables were presented as frequencies. We also compared baseline characteristics between diabetes and non-diabetes groups through Rao-Scott Chi-square test and *t* test. Logistic regression model was employed to calculate odds ratios (ORs) and 95% confidence intervals (CIs). To adjust for potential confounding effects, multivariate logistic regression analyses were conducted after adjusting for the socio-demographic characteristics and several chronic diseases. The map was drawn by MapInfo 7.0 (MapInfo Corporation, North Greenbush, NY, USA).

## 3. Results

**Figure 1:**
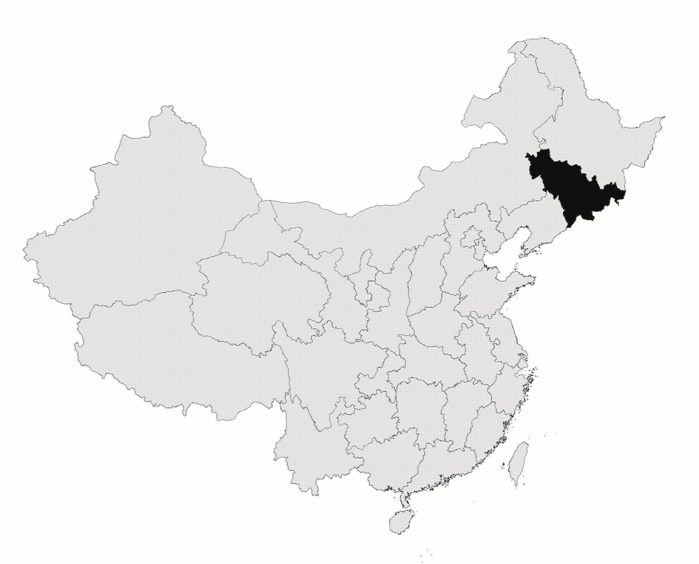
Geographical position of Jilin Province in northeast China.

All the participants in this study were recruited from general population in Jilin province, northeast China (as Figure 1 shows the location). Of the 9600 rural participants, a total of 687 (7.2%) were diagnosed with DM (7.4% in female, 6.9% in male) and the estimated prevalence of obesity in this population was 14.8%. Most subjects of this study were manual workers (67.7%) and 73.6% participants with an education below senior middle school. The Rao-sccot χ^2^ test in Table 1 shows the distribution of socio-demo characteristics between diabetes group and non-diabetes group were different in age, BMI, education, occupation, marriage, drink, smoke, exercise, family history of diabetes, blood lipids and SBP. In addition, we found the coverage of New Cooperative Medical System (NRCMS), a new kind of medical insurance popularized by Chinese government then years ago, reached 77.2%.

To examine whether the statistically significant factors found in the univariate analysis have association with DM, multivariate logistic regressions for the rate of DM were performed subsequently. As Table 2 below shows, after adjusting for potential confounding factors of socio-demographic characteristics, the result suggests a greater risk to have diabetes with the age increasing: compared with the young, the middle age group represented a nearly three-fold risk of developing DM and the elderly were under an over four-fold risk. Compared with the normal BMI group, a higher BMI was also significantly associated with diabetes (overweight: OR = 1.9810, 95% CI: 1.64, 2.39; obesity: OR = 2.63, 95% CI: 2.11, 3.28). Participants who exercise frequently were more likely than those never or rarely exercise to have DM. People with a family history of DM have over four-fold risk for developing diabetes (OR = 4.18, 95% CI: 2.64, 6.61).

Prevalence, awareness, treatment and control of the DM for the overall sample according to sex were summarized in Table 3.

Of the 687 participants with DM, about two-thirds were aware of their DM status. Of the subjects who were aware of their status, about 90% subjects have taken measures to control blood glucose, and about one-third of who have taken measures controlled their blood glucose to normal. Among participants aware of presenting with DM, Rao-scot Chi-square test suggested females had a better consciousness of their diabetes status than males, but the percentage of treatment and control presented no difference. Furthermore, we also conducted a Rao-scot Chi-square test aiming to compare the consciousness of disease surveillance between participants who were aware of their DM status and others (including participants without DM and those with DM but weren’t aware of their DM status). To identify the result more clearly, we have excluded participants with diagnosed hypotension and dyslipidemia on the basis of the original crowd. From Table 4, we observed that participants who were aware of their DM status more likely monitored their blood sugar, blood lipid and blood pressure than that in other group, and the monitoring frequency were also higher in the former group.

## 4. Discussion

With the rapid development of the economy in recent years, the lifestyle and dietary habits have changed, leading as a result to increasing prevalence of DM [13]. China is a large agricultural country, and 80 percent of its total population live in rural areas. Knowing the status of DM and health services can be very important in reducing the prevalence of this disease and improving the provision of basic medical services in rural China. The estimated prevalence of DM in Jilin Province was 8.2% in 2012 [14]. Compared with the overall prevalence of the whole country (11.6%) [7], the result of this study suggested a lower prevalence level, but still higher than other provinces and countries [15,16,17].

In agreement with some previous studies [18,19,20], our study showed the risk for diabetes increased with age, BMI and family history. Participants with higher education level had a lower risk of diabetes that coincided with previous studies [21,22]. A plausible explanation is that less-educated participants may be less cognizant of the damage caused by diabetes, and therefore may be less inclined to conduct regular disease surveillance. We also observed participants with divorced or separated marriage status had a higher risk of diabetes than that of the married group, similarly to a study which has reported that not being married, and specifically widowhood, were more consistently associated with an increased risk diabetes in men through unfavorable changes in lifestyle, diet and adiposity [23]. In addition, hypotension and dyslipidemia were found to be risk factors for diabetes in this study, as the results show that the average values of SBP, TG, TC and HDL-C in the diabetes group are significantly higher than that in the non-diabetes group, which is in line with the results of a 20 year follow-up analysis from the Framingham Offspring Study [18].

It is noticeable that this study has observed exercise was a risk factor for DM, which is different from previous studies [24]. We believe that the association between exercise and higher prevalence of DM in this study is probably related to diabetes awareness. Wang’s research indicated that the rate of treatment in previously-diagnosed DM patients was fourfold higher than in the newly-diagnosed DM group (20% *vs.* 5%). A plausible explanation is the fact that previously- diagnosed DM patients are aware of their diabetes status, they have a deeper understanding of the harmfulness of diabetes than general people. Subsequently, positive measures to slow down the progress of the disease were taken. Besides taking hypoglycemic drugs, lifestyle modifications such as forming a low-fat diet habit, regular physical exercise and quitting smoking and drinking were also put on their agendas. A Danish study reported a similar result as ours. They suggested that Type 2 DM patients reported having a healthier lifestyle. Concerning physical activity, they found increased self-reported levels among Type 2 DM patients [25]. Having a better understanding of their diabetes condition, a significant proportion of previously-diagnosed DM patients are more likely to report involvement in physical activities than the average population. This phenomenon may have led to the result. As a result of our cross-sectional design, our ability to draw causal inference is limited. However, it is indeed proved that exercise is a protective factor for diabetes [26,27].

About two-thirds (69.0%) of participants with DM were aware of their DM status, which is much lower than other countries [28,29]. Among the participants who were aware of their status of diabetes, about 12% of used no method to control their blood glucose. One plausible explanation is the lack of knowledge about chronic diseases and regular physical examination by the primary care system. The traditional Chinese concept of “Hui ji ji yi”(讳疾忌医), which means people want to conceal a disease rather than cure it, may also have an effect on this situation. However, It is worth noting that over one-third of participants who had received treatment controlled their DM condition (FBG < 6.5 mmol/L). The estimated prevalence of effective control in this study was higher compared with some prior studies [19,30]. Good patient compliance and low drug resistance may contribute to this situation, and the medical benefits of NRCMS with high coverage may also work, but further explorations are needed to better explain this issue.

In addition, our study also observed that participants with DM tended to monitor their blood pressure and blood lipid status more than those without. A possible explanation is the fact that patients with DM have medical examinations more frequently and receive more information about chronic diseases, thus they will pay more attention to their health condition. As a result of the diagnosis of DM, people with hypertension and dyslipidemia might be prompted to get regularly monitored and obtain treatment, therefore, screening for DM would allow early diagnosis and prevention of hypertension and dyslipidemia, which would benefit the prevention and control of atherosclerotic cardiovascular diseases [31]. The Chinese government and health departments should strengthen the construction of the primary health care system and take positive measures to improve the awareness of chronic diseases among the public.

A key strength of our study is the study design: a face-to-face interview based on a large population. The use of complex weighted computation makes the sample more representative. The precise physical measurements improved the validity of the results. Nonetheless, there are still some limitations that should be mentioned. First, the awareness of DM was based on self-reported information. Second, we didn’t distinguish Type 1 and Type 2 diabetes although Type 2 diabetes is the predominant type of diabetes in adults. Also, the measurement of blood sugar levels may limit the accuracy of the result. Finally, the nature of cross-sectional might limit our ability to address the “chicken-egg” problem such as the association between physical exercise and DM.

## 5. Conclusions

To sum up, we observed a high prevalence and low awareness status of DM among the rural residents in Jilin Province, but the rate of effective control in those who have received treatment was considerable. DM was positively associated with age, BMI, hypotension, dyslipidemia and was high in participants with a family history of diabetes and those who exercise frequently, but low for high education level and married participants. The low rate of disease surveillance should draw health authority’s attention.

## Figures and Tables

**Table 1:** Comparison of socio-demographic characteristic between diabetes group and non-diabetes group.

Characteristic	Subcategory	Total (*n* = 9600, 100.0%)	Subjects with Diabetes (*n* = 687, 7.2%)	Subjects without Diabetes (*n* = 8913, 92.8%)	χ^2^/t	*p*
Gender	Women	4716 (49.1)	348 (50.7)	4368 (49.0)	0.78	0.389
	Men	4884 (50.9)	339 (49.3)	4545 (51.0)		
Age	Young (18–44)	5286 (56.1)	169 (24.5)	5217 (58.5)	362.87	<0.001
	Middle (44–59)	2782 (29.0)	293 (42.6)	2489 (27.9)		
	Old (60–80)	1433 (14.9)	226 (32.9)	1207 (13.5)		
BMI	Normal	4454 (47.6)	201 (29.5)	4252 (49.0)	166.40	<0.001
	Underweight	568 (6.1)	18 (2.7)	549 (6.3)		
	Overweight	2878 (31.8)	300 (43.8)	2677 (30.9)		
	Obesity	1355 (14.5)	164 (24.0)	1191 (13.7)		
Education	Primary school and below	3722 (38.8)	363 (52.8)	3359 (37.7)	77.64	<0.001
	Junior middle school	3340 (34.8)	173 (25.1)	3167 (35.5)		
	Senior middle school	1567 (16.3)	113 (16.5)	1453 (16.3)		
	Under graduate and above	972 (10.1)	38 (5.6)	933 (10.5)		
Occupation	Intelligence	1685 (17.6)	86 (12.5)	1599 (17.9)	116.60	<0.001
	Manual	6480 (67.5)	440 (64.0)	6040 (67.8)		
	Retired	306 (3.2)	66 (9.5)	240 (2.7)		
	Others	1130 (11.8)	96 (13.9)	1034 (11.6)		
Marriage	Married/cohabitation	8227 (85.7)	620 (90.3)	7607 (90.3)	86.26	<0.001
	Single	939 (9.8)	9 (1.3)	930 (10.4)		
	Divorced/Separated	93 (1.0)	13 (7.3)	80 (0.9)		
	windowed	341 (3.6)	45 (6.6)	296 (3.3)		
Drink	Yes	3313 (67.6)	190 (27.7)	2923 (32.7)	8.15	0.006
	No	6487 (32.4)	497 (72.3)	5990 (67.2)		
Smoke	Never	5696 (59.3)	401 (58.4)	5295 (59.4)	27.49	<0.001
	Now	3188 (33.2)	202 (29.4)	2985 (33.5)		
	Once	716 (7.5)	84 (8.1)	632 (7.1)		
Exercise	Never or rare	5405 (56.3)	314 (45.6)	5092 (57.1)	118.47	<0.001
	Sometimes	2099 (21.9)	114 (16.6)	1985 (22.3)		
	Frequently	2096 (21.8)	260 (37.8)	1837 (20.6)		
Family history	Yes	1080 (11.2)	188 (27.4)	891 (10.0)	206.53	<0.001
	No	8520 (88.8)	499 (72.6)	8020 (90.0)		
Blood lipid ^**a**^	TG	2.03 ± 1.82	3.02 ± 2.92	1.92 ± 1.61	17.53	<0.001
	TC	4.90 ± 1.10	5.32 ± 1.34	4.86 ± 1.61	12.08	<0.001
	LDL-C	2.90 ± 0.89	3.11 ± 1.01	2.88 ± 0.87	7.39	<0.001
	HDL-C	1.40 ± 0.39	1.25 ± 0.34	1.42 ± 0.40	−11.88	<0.001
Blood pressure ^**a**^	SBP	132.29 ± 21.77	140.20 ± 23.48	131.35 ± 21.10	12.09	<0.001
	DBP	80.01 ± 11.79	82.50 ± 11.73	79.69 ± 11.60	0.49	0.194
Blood sugar ^**a,b**^	FPG	5.41 ± 1.89	8.78 ± 3.70	5.00 ± 0.93	72.74	<0.001

**^a^** *t*-test was used to analysis the difference between the two groups. **^b^** Blood sugar was measured by Bayer Bai Ankang fingertip blood glucose monitor machine after all the participants fast of 10 h or more overnight.

**Table 2:** Multivariate logistic regression analysis of the factors associated with prevalence of diabetes.

Category	Subcategory	OR	95% CI	*p*
Age	Young (18–44)	1		<0.001
	Middle (44–59)	3.37	1.85–2.99	
	Old (60–80)	4.51	2.54–4.48	
BMI	Normal	1		<0.001
	Underweight	1.10	0.62–1.94	
	Overweight	1.55	1.28–1.88	
	Obesity	1.76	1.39–2.24	
Education	Primary school and below	1		0.008
	Junior middle school	0.72	0.58–0.89	
	Senior middle school	0.82	0.64–1.06	
	Under graduate and above	0.58	0.38–0.88	
Marriage	Married/cohabitation	1		0.005
	Single	0.34	0.15–0.74	
	Divorced/Separated	1.86	1.00–3.45	
	windowed	0.85	0.61–1.18	
Exercise	Never or rarely	1		<0.001
	sometimes	1.29	0.97–1.70	
	frequently	1.71	1.42–2.07	
Family history	No	1		<0.001
	Yes	3.68	2.97–4.54	
Diagnosed hypotension	No	1		<0.001
	yes	1.60	1.35–1.91	
Diagnosed dyslipidemia	No	1		<0.001
	yes	2.28	1.92–2.708	

**Notes:** Statistical analysis by forward stepwise logistic regression with a 0.10 significance level for removal from and a significance level of 0.05 for addition to the model. The variables studied were age (three groups), BMI (normal, underweight, overweight, obesity), education (four groups), marriage (three groups), exercise (three groups), family history (yes/no), diagnosed hypotension (yes/no), diagnosed dyslipidemia (yes/no). Results are expressed as odds ratio and 95% confidence interval).

**Table 3:** Prevalence, awareness and treatment of diabetes, overall and by sex.

Characteristic	All (N)	Female (n, %)	Male (n, %)	*χ*^2^ (*p*)
All subjects	9600	4716 (48.1)	4884 (50.9)	
Diabetes	687 (7.2)	348 (7.4)	339 (6.9)	0.74 (0.39)
Subjects with diabetes	687	348	339	
Aware	474 (69.0)	270 (77.6)	204 (60.2)	26.74 (<0.001)
Subjects aware of diabetes	474	270	204	
Treated	418 (88.2)	243 (90.0)	175 (85.8)	2.26 (0.13)
Subjects treated for diabetes	418	243	175	
Fasting plasma glucose < 7 mmol/L	178 (42.6)	106 (43.6)	72 (41.1)	0.86 (0.35)
Fasting plasma glucose < 6.5 mmol/L	144 (34.4)	84 (34.6)	60 (34.3)	0.24 (0.62)

**Notes:** diabetes was considered to be controlled if FPG ≤ 6.5 mmol/L.

**Table 4:** Comparison of surveillance of blood pressure and blood lipid between participants with DM awareness and other group.

Characteristic	Subcategory	Awareness of DM (n, %)		Others (n, %)		*p*
Total		191	100	1231	100	
Monitor blood pressure	Within a month	130	68.2	708	57.5	0.012
	Over a month	55	28.9	450	36.6	
	Never	1	0.8	28	2.3	
	Not clear	4	2.1	45	3.7	
Monitor blood lipid	Within a month	29	15.3	75	8.0	<0.001
	Over a month	77	40.1	406	33.0	
	Never	72	37.5	686	55.7	
	Not clear	13	7.0	64	5.2	
Monitor blood glucose	Within a month	117	61.1	126	10.2	<0.001
	Over a month	67	35.0	421	34.2	
	Never	5	2.5	629	51.1	
	Not clear	3	1.4	55	4.4	

## Abbreviations

The following abbreviations are used in this manuscript:

DM	diabetes mellitus
IDF	International Diabetes Federation
NCMS	New Rural Cooperative Medical System
FBG	fasting blood glucose
BMI	Body mass index
TG	triglyceride
TC	cholesterin
HDL-C	high density lipoprotein cholesterol
LDL-C	low density lipoprotein-cholesterol
OR	odds ratios
CI	confidence interval
SBP	systolic blood pressure
DBP	diastolic blood pressure
